# *Plasmodium knowlesi* from archival blood films: Further evidence that human infections are widely distributed and not newly emergent in Malaysian Borneo

**DOI:** 10.1016/j.ijpara.2009.03.003

**Published:** 2009-08

**Authors:** Kim-Sung Lee, Janet Cox-Singh, George Brooke, Asmad Matusop, Balbir Singh

**Affiliations:** a Malaria Research Centre, Faculty of Medicine and Health Sciences, Unversiti Malaysia Sarawak, 93150 Kuching, Sarawak, Malaysian Borneo, Malaysia; b Sarawak State Health Department, Kuching, Sarawak, Malaysian Borneo, Malaysia

**Keywords:** *Plasmodium knowlesi*, Malaria, Epidemiology, Archival blood films, Nested-PCR

## Abstract

Human infections with *Plasmodium knowlesi* have been misdiagnosed by microscopy as *Plasmodium malariae* due to their morphological similarities. Although microscopy-identified *P. malariae* cases have been reported in the state of Sarawak (Malaysian Borno) as early as 1952, recent epidemiological studies suggest the absence of indigenous *P. malariae* infections. The present study aimed to determine the past incidence and distribution of *P. knowlesi* infections in the state of Sarawak based on archival blood films from patients diagnosed by microscopy as having *P. malariae* infections. Nested PCR assays were used to identify *Plasmodium* species in DNA extracted from 47 thick blood films collected in 1996 from patients in seven different divisions throughout the state of Sarawak. *Plasmodium knowlesi* DNA was detected in 35 (97.2%) of 36 blood films that were positive for *Plasmodium* DNA, with patients originating from all seven divisions. Only one sample was positive for *P. malariae* DNA. This study provides further evidence of the widespread distribution of human infections with *P. knowlesi* in Sarawak and its past occurrence. Taken together with data from previous studies, our findings suggest that *P. knowlesi* malaria is not a newly emergent disease in humans.

## 1 Introduction

*Plasmodium knowlesi*, a malaria parasite of Old World monkeys (Garnham, 1966), is one of the five malaria species known to cause human malaria (Cox-Singh and Singh, 2008). Following our report of a large focus of human *P. knowlesi* infections in the Kapit division of Sarawak, Malaysian Borneo (Singh et al., 2004), cases of naturally acquired human infections with *P. knowlesi* have been reported from many areas in Southeast Asia including Thailand (Jongwutiwes et al., 2004), Myanmar (Zhu et al., 2006), the Philippines (Luchavez et al., 2008), Singapore (Ng et al., 2008), Sabah State, Malaysian Borneo (Cox-Singh et al., 2008) and Peninsular Malaysia (Cox-Singh et al., 2008; Vythilingam et al., 2008).

*Plasmodium knowlesi* malaria in humans is routinely misdiagnosed by microscopy as *Plasmodium malariae* malaria due to the morphological similarities between the two species and the only reliable diagnostic method to correctly distinguish between the two species is two nested PCR assays (Cox-Singh and Singh, 2008). Our prospective molecular epidemiological studies conducted on 960 samples collected from malaria patients in Sarawak between 2000 and 2006 showed that by using nested PCR assays 266 were diagnosed as *P. knowlesi* and only four were *P. malariae* cases, although 312 had been diagnosed as *P. malariae* by microscopy (Singh et al., 2004; Cox-Singh et al., 2008). All four *P. malariae* infections had been acquired by logging camp workers who had recently returned from malaria-endemic countries. The apparent lack of indigenous *P. malariae* cases in Sarawak raised the question of whether previous malaria cases identified by microscopy as *P. malariae* were *P. knowlesi* malaria. Here, we present further evidence of the past incidence and widespread distribution of human *P. knowlesi* infections in the state of Sarawak based on a retrospective study using DNA extracted from archival malaria blood films diagnosed as *P. malariae* by microscopy.

## 2 Materials and methods

### 2.1 Malaria blood films

**Fig. 1:**
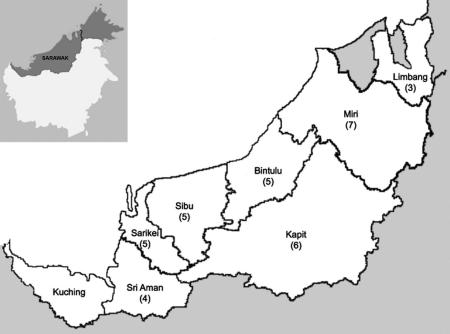
Map of Sarawak (Malaysian Borneo) showing the number of *P. knowlesi* malaria cases detected in each of the seven administrative divisions from which samples were collected, with the inset showing the location of Sarawak on the island of Borneo. The numbers of single and mixed *Plasmodium knowlesi* cases detected by PCR for each division are in brackets.

A total of 47 thick blood films prepared in 1996 from malaria patients diagnosed by microscopy as having *P. malariae* from seven administrative divisions in the state of Sarawak were obtained from the Parasitology Diagnostic Laboratory of the Sarawak State Health Department. They had been collected at hospitals and health clinics in the following administrative divisions; Sri Aman, Sarikei, Sibu, Bintulu, Kapit, Miri and Limbang (Fig. 1). Malaria blood films collected in 1996 were the oldest samples that we were able to obtain at the time of this study. A total of 392 blood films diagnosed as *P. malariae* were received by the Parasitology Diagnostic Laboratory during that year for confirmation of the infecting *Plasmodium* sp. Of these, 47 blood films were selected at random with the only selection criteria being that they were not collected from patients at the same hospital or health clinic in each administrative division. These blood films were sent to the Malaria Research Centre, Universiti Malaysia Sarawak (UNIMAS) for DNA extraction and examination by PCR.

### 2.2 DNA extraction

DNA was extracted from all 47 blood films by the phenol–chloroform method as described previously (Cox-Singh et al., 2008). Briefly, each blood film was moistened with Tris–EDTA (TE) buffer, scraped and collected in a microcentrifuge tube containing 100 μl TE buffer. Into each tube was added 10 μl 10 mg/ml Proteinase K and 100 μl of lysis buffer (5 mM EDTA, 0.5% sodium dodecylsulfate, 200 mM NaCl and 100 mM Tris–Cl, pH8), and incubated in a thermomixer at 56 °C with shaking at 900 rpm for 10 min. DNA was purified by adding an equal volume of phenol–chloroform–isoamyl alcohol followed by vigorous mixing for 15 s and centrifugation for 2 min at 20,800*g*. The aqueous phase was transferred into a new microcentrifuge tube and the organic phase was re-extracted by adding 100 μl TE buffer and mixing, then centrifuging in a similar manner. Aqueous phase from the second extraction was pooled with that from the first and DNA was ethanol precipitated as previously described (Ausubel et al., 2003). The DNA pellet was dissolved in 50 μl TE buffer. Positive control thick blood films, at parasitaemias of 310 and three parasites per μl of blood prepared from *Plasmodium falciparum* clone K1 cultured in vitro, and negative control thick blood films prepared from uninfected blood, were also included during DNA extraction. Each archival blood film was processed individually together with two positive (*P. falciparum*) and one negative control as a precaution to prevent cross-contamination between archival slides during DNA extraction.

### 2.3 Nested PCR

DNA samples were initially analysed using *Plasmodium* genus and species-specific nested PCR assays as described previously (Singh et al., 2004). The first PCR amplification (nest 1) using genus-specific primers for each sample was carried out in a 50 μl reaction mixture containing 2.5 mM of each primer (rPLU1 and rPLU5), 1× PCR buffer (50 mM KCl, 10 mM Tris–HCL), 3 mM MgCl2, 200 mM each deoxynucleotide triphosphate, 1.25 U of Taq DNA polymerase (Promega, USA) and 15 μl of DNA template. PCR amplification was performed using the following conditions; 94 °C for 4 min, 35 cycles of 94 °C for 30 s, 55 °C for 1 min and 72 °C for 1 min, followed by 72 °C for 4 min. Two microlitres of nest 1 amplification was used as template DNA in the second PCR amplification (nest 2). Nest 2 PCR amplification was carried out in a 20 μl reaction mixture containing similar concentrations of species-specific primers and other constituents, except that 2 mM MgCl_2_ and 0.5 U Taq were used. Conditions for nest 2 PCR amplification were similar to those of nest 1, except for the annealing temperature which was 62 °C for *Plasmodium* genus-specific primers, 58 °C for four human *Plasmodium* species-specific primer pairs (rFAL1/rFAL2, rVIV1/rVIV2, rMAL1/rMAL2 and rOVA1/rOVA4) (Snounou et al., 1993; Davis et al., 2001) and 60 °C for *P. knowlesi*-specific primers (Pmk8/Pmkr9) (Singh et al., 2004). Nest 2 PCR products were analysed by gel electrophoresis and ethidium bromide staining. Precautions to prevent cross-contamination in nested PCR assays were taken as described previously (Singh et al., 2004).

## 3 Results

Analysis by nested PCR assay revealed that 36 (76.6%) of 47 blood films were positive with *Plasmodium*-specific primers (Table 1). Subsequent analysis of these 36 samples with species-specific nested PCR assays showed that 35 (97.2%) had *P. knowlesi* DNA and one (2.8%) contained *P. malariae* DNA. Twenty-nine were single *P. knowlesi* infections and six were *P. knowlesi* mixed with *Plasmodium vivax*. Eleven (23.4%) of 47 DNA samples examined were negative for *Plasmodium* DNA although these samples were from patients with parasite counts ranging from 583 to 4,826 (geometric mean = 1,400) parasites per μl blood and eight (72.7%) of those had counts greater than 1,000 parasites per μl blood. The 36 blood films that tested positive for *Plasmodium* DNA had parasite counts between 360 and 32,280 (geometric mean = 2,286) parasites per μl blood and the counts were not significantly different from those that tested negative by PCR (mean ratio = 1.62; 95% Confidence Interval (CI) 0.93–2.81; *P* = 0.085). Despite the negative PCR results with some of the archival samples, we were able to consistently amplify *Plasmodium* DNA from *P. falciparum*-positive control blood films with parasite counts of 310 and three parasites per μl of blood, while no amplification was observed for negative controls. The 35 PCR-positive blood films for *P. knowlesi* had originally been collected from patients from seven divisions throughout the state of Sarawak (Fig. 1).

## 4 Discussion

Malaria blood films have been shown to be a useful source of DNA for molecular epidemiological studies (Kimura et al., 1995; Edoh et al., 1997; Scopel et al., 2004; Cox-Singh et al., 2008). Here we use archival blood films to demonstrate the presence of *P. knowlesi* malaria in seven of the administrative divisions of Sarawak in 1996, long before *P. knowlesi* was recognised as a significant human pathogen in this region. Detection of malaria parasite DNA extracted from archival slides was specific, but sensitivity was variable and independent of parasitaemia. A similar finding of low sensitivity of nested PCRs for malaria using DNA extracted from thick blood films in an epidemiological study has been reported (Scopel et al., 2004). As in our study, there was no statistical difference between the parasite counts in blood films that tested positive by PCR (10–4,000 parasites per μl) and those that did not (20–800 parasites per μl) (Scopel et al., 2004). The blood films we examined had been stored for more than 10 years in conditions not designed to protect DNA integrity. Other studies have shown that DNA degradation may occur in Giemsa-stained blood films stored for greater than 4 years (Yokota et al., 1995) and that oxidative damage by atmospheric oxygen may cause DNA degradation in dried tissues (Matsuo et al., 1995).

In our study only one blood film was PCR-positive for *P. malariae.* This slide was from a patient who was working in a logging camp in the Kapit division. Our recent studies have shown that out of 312 malaria cases diagnosed by microscopy as *P. malariae*, only four *P. malariae* cases were detected by nested PCR assays and all four patients were logging camp workers who had returned recently to the Kapit division after working in malaria-endemic countries overseas (Cox-Singh et al., 2008). However, there was no information on the travel history of the first individual and we could not determine the origin of his infection.

The first recorded malaria survey in the state of Sarawak that involved microscopy for identification of malaria parasites was conducted from July to November 1952, by a team from the World Health Organization (de Zulueta, 1956). They found that of the 421 malaria infections detected during screening of communities in six areas, 173 (41.1%) were *P. falciparum,* 142 (33.7%) were *P. malariae* and 106 (25.2%) were *P. vivax.* In that study, *P. malariae* was the predominant species detected in the Kuching division (known as the 1st Division in the 1950s) and the Miri and Bintulu divisions (previously known as the 4th Division), where it accounted for 76.3% and 68.8% of 180 and 136 malaria infections detected, respectively. Furthermore, entomological observations conducted in that survey showed that *Anopheles leucosphyrus*, which is capable of transmitting *P. knowlesi* (Warren and Wharton, 1963; Collins et al., 1967), was the main malaria vector in Sarawak (de Zulueta, 1956). *Anopheles latens* (previously known as *An. leucosphyrus* A (Baimai et al., 1988; Sallum et al., 2005)), has been incriminated as the vector for *P. knowlesi* in the Kapit division (Vythilingam et al., 2006). The only way to prove that the *P. malariae* reported by Zueleta were *P. knowlesi* is by PCR examination of blood smears collected during that survey. These *P. malariae* smears are not available but there is a high level of suspicion that at least some of these were *P. knowlesi*, given that the predominant vector in 1952 was one capable of transmitting *P. knowlesi* and based on the current lack of evidence of indigenous cases of *P. malariae* in Sarawak. It is highly unlikely that in the interim period malaria control methods designed to block human-to-human transmission of malaria, through the provision of insecticide-impregnated bed nets and residual insecticide spraying of houses, would have had any significant impact on zoonotic *P. knowlesi* malaria transmission or led to the specific disappearance of *P. malariae* but not *P. falciparum* or *P. vivax* in Sarawak. Furthermore, the lifestyle of the rural communities in Sarawak, centered on subsistence farming and other activities associated with the forests and forest-fringe, has remained unchanged for decades. It is therefore probable that at least some of the microscopy-identified *P. malariae* cases from the 1952 malaria survey were *P. knowlesi* infections.

The morphological similarities between *P. knowlesi* and *P. malariae* make identification of *P. knowlesi* by microscopy extremely difficult. Prior to the development of PCR assays and *P. knowlesi*-specific primers (Singh et al., 2004), the only available method to correctly identify *P. knowlesi* was by observing the pathological outcome after injecting infected blood into rhesus monkeys (*Macaca mulatta*) as *P. knowlesi* parasites cause lethal infections in these monkeys (Chin et al., 1965, 1968; Garnham, 1966). Such a method was expensive and restricted to research institutes, making it impractical for use in routine diagnostic laboratories. The current study, utilising molecular detection methods, demonstrates that human infections with *P. knowlesi* were widely distributed in 1996 and that they have passed unrecognised in Sarawak for many years. Rural populations in Sarawak traditionally use the forest as a food and material resource. The macaque host, mosquito vector and parasite transmission cycle are well established in the jungles of Sarawak and in view of the current prevalence of *P. knowlesi* malaria in the human population in Sarawak (Cox-Singh et al., 2008), it seems highly likely that *P. knowlesi* in humans is not a newly emergent disease, but rather a zoonosis masked until very recently by morphological similarities with *P. malariae*.

## Figures and Tables

**Table 1:** Results of nested PCR assays for detection of *Plasmodium* species in blood films collected in 1996.

Division	Total no. of slides examined	Parasitaemia (geometric mean [range])	No. *Plasmodium* DNA positive	Species-specific nested PCR					
				Pf	Pv	Pm	Po	Pk	Pv and Pk
Miri	14	1,103 [530–3,888]	7	0	0	0	0	6	1
Bintulu	7	2,044 [360–4,826]	5	0	0	0	0	4	1
Sibu	7	2,673 [720–8,240]	5	0	0	0	0	5	0
Kapit	7	1,156 [470–6,400]	7	0	0	1	0	6	0
Sarikei	5	3,798 [1,800–18,000]	5	0	0	0	0	3	2
Sri Aman	4	10,766 [6,400–32,280]	4	0	0	0	0	2	2
Limbang	3	6,563 [4,800–9,440]	3	0	0	0	0	3	0
Total	47		36	0	0	1	0	29	6

Pf, *Plasmodium falciparum*; Pm, *Plasmodium malariae*; Pv, *Plasmodium vivax*; Po, *Plasmodium ovale*; Pk, *Plasmodium knowlesi*.
